# Mathematical Model Construction of the Bionic Irregular Surface of Turtle Shell

**DOI:** 10.1155/abb/5546243

**Published:** 2026-05-13

**Authors:** Sanling Fu, Zijun Chen, Tianhua Chen, Yafei Wang, Fu Zhang

**Affiliations:** ^1^ College of Physical Engineering, Henan University of Science and Technology, Luoyang, China, haust.edu.cn; ^2^ College of Agricultural Equipment Engineering, Henan University of Science and Technology, Luoyang, 471003, China, haust.edu.cn; ^3^ College of Biological and Agricultural Engineering, Jilin University, Changchun, 130025, China, jlu.edu.cn; ^4^ School of Agricultural Engineering, Jiangsu University, Zhenjiang, 212013, China, ujs.edu.cn

**Keywords:** bionics, reverse engineering, transplanter floating plate, turtle abdominal armor surface

## Abstract

The irregular surface morphology of the turtle shell can be applied to the design of the floating plate of a rice transplanter to mitigate the serious problems of mud and water resistance. An adult Brazilian turtle was selected as the research object in this study. A 7‐axis absolute arm measuring machine and Geomagic Studio software were used to acquire and process point cloud data of the turtle’s plastron. Based on the structural characteristics of the turtle shell, four curved surfaces with dense point cloud distributions were segmented, filtered using CATIA software, and exported as three‐dimensional coordinate data. MATLAB was used to perform polynomial fitting of the three‐dimensional point cloud data. The fitting equations for the four surfaces, as well as the sum of squared errors (SSEs), root mean square error (RMSE), and coefficient of determination (*R* ^2^), were obtained. The results showed that the maximum relative errors between the fitted and actual values for the front, rear, and side models were 8.96%, 9.36%, and 5.86%, respectively. The corresponding mean relative errors were 4.58%, 4.67%, and 2.98%, respectively. These mean relative errors fall within the ±5% tolerance permitted in engineering design, thereby verifying the validity of the models and enabling the transformation of the turtle plastron surface from a biological form into a mathematical model. This study provides a theoretical foundation for the bionic application of the turtle plastron surface morphology and offers a reference for the bionic design of floating plates for rice transplanters.

## 1. Introduction

China possesses vast and diverse cultivable land resources, with paddy fields accounting for approximately one‐quarter of the nation’s total arable land [1–4]. With advances in agricultural equipment and technology, rice transplanting machinery has attracted increasing research attention from scholars [5–7]. As a critical component of the paddy rice transplanter, the floating board plays an essential role in maintaining operational stability, leveling the field surface, and ensuring an appropriate transplanting depth [8–10]. In addition, owing to the complex paddy‐field environment, contact between the floating plate and the mud generates sliding resistance during transplanter operation. This interaction generates waves and mud–water disturbances, which increase tractive resistance, reduce traveling speed, damage transplanted seedlings, and increase energy consumption [11–14]. Therefore, reducing the sliding resistance between the floating plate and the mud during transplanter movement can improve both operational efficiency and working quality [15–17].

Despite these advances, existing engineering solutions still exhibit several technical limitations. Traditional floating plate designs primarily rely on simplified geometric modifications or the incorporation of external components. Such added devices inevitably increase the overall weight, structural complexity, and energy consumption of the transplanter and are also prone to severe wear, structural failure, and high maintenance requirements in abrasive mud–water environments [18]. Simple flat or regularly curved plates fail to adapt adequately to the complex, nonlinear multiphase flow of mud and water. This can result in localized stress concentrations, continuous mud accumulation (i.e., the bulldozing effect), and suboptimal drag‐reduction performance [19, 20]. To address the long development cycle and high design cost of traditional floating plates, Tang et al. [21] employed virtual prototyping technology to optimize plate shape and thickness and established a virtual model for simulation analysis. The optimal performance was achieved when the floating board thickness was 60 mm. During machine operation, the floating plate was able to smooth the field surface while simultaneously performing ditching and ridging functions. To address the problem that the rolling motion of the ditching and leveling device carried mud and water outward during operation of the rice direct seeder, thereby washing away adjacent seeds, Wang et al. [22] designed a front‐end with a straight‐chamfered structure. A wooden anti‐wave device was installed on both sides of the direct seeder frame to break mud flow and provide diversion buffering, thereby reducing forward resistance and improving operational stability during mud breaking. To address the mud accumulation problem in rice transplanters, Zhang and Liang [23] used the integral floating plate as the research object, established a mathematical model of the viscous flow field with a free surface around the plate, and conducted simulation analyses to identify the main sludge‐forming regions. They further clarified the main locations and causes of sludge formation and proposed an optimization scheme for the mud‐control structure of the floating plate.

As one of the most ancient vertebrate lineages on Earth, turtles exhibit highly evolved body structures, forms, and functions [24, 25]. This evolutionary refinement has enabled turtles to survive successfully under the natural law of survival of the fittest. Based on analyses of turtle shell surface characteristics, previous studies have investigated its mechanical properties, load‐bearing mechanisms, and internal pore structure, and have developed laminated composite materials inspired by these features [26–28]. However, few studies have focused on the surface morphology of turtle shells. Inspired by natural biological structures, this study selects the semiaquatic, semiterrestrial Brazilian turtle as a bionic prototype and, in combination with engineering bionics, investigates the bionic morphology of its plastron for application to the floating board of a rice transplanter. This approach is of great significance for reducing the sliding resistance of the floating board of the paddy rice transplanter and improving its operational efficiency.

Previous studies have confirmed that reducing soil adhesion not only directly lowers sliding resistance but also decreases the traction drag of agricultural machinery [18–20, 29, 30]. In this context, the turtle‐shell‐based mathematical model provides a theoretical basis for the biomimetic optimization of floating plates, ensuring that drag reduction is systematically integrated into the design process rather than achieved through empirical trial‐and‐error adjustments.

This study established a mathematical model of the turtle plastron surface, thereby transforming a biological prototype into design‐ready parameters. The results demonstrate that the established models can be directly applied to the biomimetic optimization of floating plates in rice transplanters. Specifically, based on the polynomial equations describing the anterior, central, and lateral regions of the shell, the bow plate, the bottom surface of the floating plate, and the ridge plate can be redesigned to conform to the surface characteristics of the turtle shell, thereby reducing drag during forward motion.

## 2. Reconstruction of Turtle Plastron Surface Model

### 2.1. The Geometric Model of the Turtle Plastron Surface

**Figure 1:**
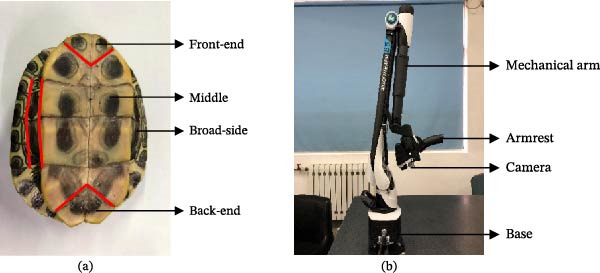
Experimental materials and equipment. (a) Brazilian turtle and (b) 7‐axis absolute arm measuring machine.

To establish a geometric model of the curved surface of the turtle plastron, a Brazilian turtle older than 4 years was selected as the research subject. To facilitate scanning, the turtle shell surface was washed with water and allowed to dry naturally, as shown in Figure 1a. To obtain three‐dimensional point‐cloud data from the surface of the Brazilian turtle’s plastron, the turtle was placed on a test bench and scanned using an Absolute Arm 85 Series 7‐axis measuring machine equipped with trigger‐probe measurement and laser‐scanning functions, manufactured by Hexagon Measurement Technology (Qingdao) Co., Ltd. The measuring machine is shown in Figure 1b.

**Figure 2:**
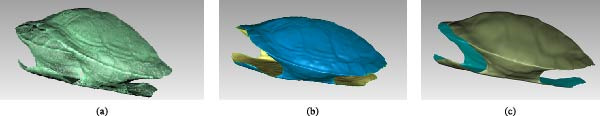
Geometric modeling of turtle shell. (a) Point cloud data, (b) polygon, and (c) accurate surface.

The scanned three‐dimensional point‐cloud data of the turtle shell are shown in Figure 2a. Because the 7‐axis Absolute Arm measuring machine also captures surrounding objects during the scanning process, the acquired model contains noise points and external isolated outliers; therefore, the turtle‐shell 3D point‐cloud data required further processing. The 3D point‐cloud model was saved in .ASC format and imported into Geomagic Studio software. Noise points and isolated outliers were removed, after which the data were encapsulated and converted into a triangular surface model. The Mesh Doctor function was then used to automatically identify and repair defects through operations including sharp‐point removal, defeaturing, mesh relaxation, and sharpening. The processed result is shown in Figure 2b. The polygon‐processed model was then converted into a precise surface, after which operations such as surface generation and grid processing were performed. Ultimately, an accurate surface model of the turtle shell point‐cloud data was obtained, as shown in Figure 2c.

**Figure 3:**
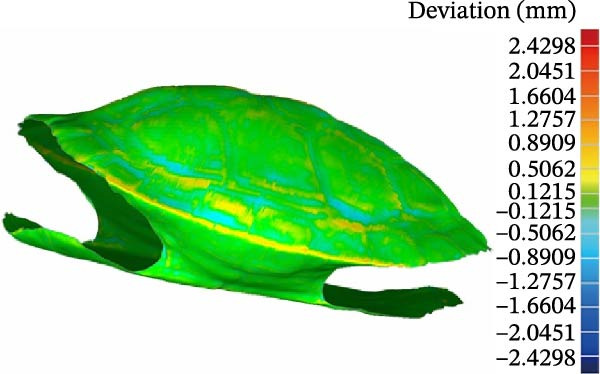
Result of deviation analysis.

Deviation analysis was then performed on the model. The results are presented in Figure 3. The maximum three‐dimensional deviation ranged from −2.4298 to +0.9456 mm, the mean deviation ranged from −0.0581 to +0.0730 mm, and the standard deviation was 0.1038 mm.

**Figure 4:**
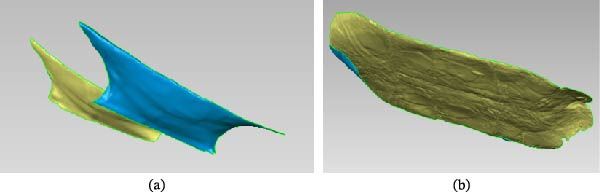
Result of turtle’s shell segmentation. (a) Side of a turtle’s shell and (b) base of a turtle’s shell.

Analysis of Figure 3 indicates that the deviation of the turtle shell model is mainly concentrated within the range of −0.1215 to +0.1215 mm. A smaller portion falls within the ranges of +0.5062 to +0.8909 mm and −0.5062 to −0.8909 mm, whereas only a very limited region, located in the upper shell area, exhibits deviations between +0.8909 and +1.2757 mm. In the biomimetic design, the bottom and lateral regions of the turtle shell were the primary focus. For the bottom shell of the turtle used in the experiment, the dimensions corresponding to the model deviation were 115.73 mm in length, 61.42 mm in width, and 4.32 mm in thickness. For the lateral shell region, the corresponding dimensions were 42.24 mm in length, 13.16 mm in width, and 5.58 mm in thickness, all of which fell within the allowable engineering design error range of ±5%. Therefore, the processed geometric model of the turtle plastron surface satisfies the requirements for engineering biomimetic design. The overall turtle shell is box‐shaped and consists of an arched carapace, a flat plastron, and bridge structures connecting the carapace and plastron. The anterior end of the plastron is blunt, whereas the posterior end contains a triangular notch. The central region is divided into eight plastral scutes, and the left and right sides are bilaterally symmetrical. When viewed from the ventral side of the plastron, the four central sections are rectangular, whereas the two terminal sections are fan‐shaped. The bridge structures on both sides curve upward in a wing‐like form. Therefore, according to the structural characteristics of the turtle shell, the curve‐cutting function in Geomagic Studio was used to separate the bottom and lateral regions of the shell. The results are shown in Figure 4.

### 2.2. Point Cloud Processing of the Turtle Plastron Surface

According to the symmetrical structural and morphological characteristics of the turtle shell bottom, one half of the plastron (i.e., the left side) was divided into three regions: the anterior, middle, and posterior regions. The surface models of these three regions were then converted into point‐cloud data in Geomagic Studio and saved as .igs files. The point‐cloud data of these three regions were imported into the Digitized Shape Editor module of CATIA, where the surface point clouds were filtered. The filtered point‐cloud data were exported in .txt format as three‐dimensional coordinate values.

**Figure 5:**
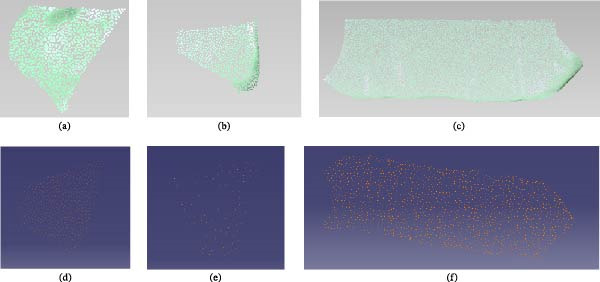
Comparison diagram of three curved point clouds at the bottom of the turtles shell. (a) Front‐end at the bottom of the turtle’s shell before filtering, (b) back‐end at the bottom of the turtle’s shell before filtering, (c) middle at the bottom of the turtle’s shell before filtering, (d) front‐end at the bottom of the turtle’ s shell after filtering, (e) back‐end at the bottom of the turtle’s shell after filtering, and (f) middle at the bottom of the turtle’s shell after filtering.

Nonuniform filtering was performed on the three surfaces. The point clouds before and after filtering are shown in Figure 5. Figure 5a–c show the point‐cloud distributions of the three surfaces before filtering. The numbers of data points were 1667, 875, and 1854, respectively. Figure 5d–f show the point‐cloud distributions of the three curved surfaces after filtering. The corresponding numbers of data points were 475, 143, and 1854, respectively. Although the amount of filtered point‐cloud data was substantially reduced, the point‐cloud images indicate that the surface features of the three filtered regions remained clear and that their overall shapes were not affected.

**Figure 6:**
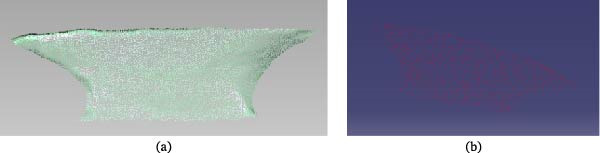
Comparison diagram of side point clouds of the turtle’s shell. (a) Side of the turtle’s shell before filtering and (b) side of the turtle’s shell after filtering.

According to the structural characteristics of the turtle shell, the lateral region was extracted, the point‐cloud data were filtered, and the three‐dimensional coordinate values were obtained. The point clouds of the lateral region before and after filtering are shown in Figure 6. Figure 6a,b show the point‐cloud data before and after filtering, respectively. The numbers of data points were 6490 and 1362, respectively. The point‐cloud image indicates that the characteristic features of the filtered surface remained clearly identifiable.

## 3. Mathematical Model of the Turtle Shell Surface

The bottom and lateral surfaces of the turtle shell do not conform well to a fixed mathematical model in terms of morphology; therefore, a polynomial fitting method was adopted. MATLAB provides a range of toolboxes, and the Curve Fitting Toolbox was used to perform polynomial fitting of the filtered point‐cloud data through the surface fitting tool (cftool).

### 3.1. The Mathematical Model of the Turtle Shell Front‐End Surface

The point‐cloud data from the left side of the turtle plastron were imported into MATLAB. The *x* and *y* coordinates were treated as independent variables, whereas the *z* coordinate was treated as the dependent variable, and polynomial functions were used to fit the surface point‐cloud features. In the cftool module of MATLAB, polynomial fitting was selected, and the orders of both *x* and *y* were specified simultaneously. In general, increasing the polynomial orders of *x* and *y* improves the fitting accuracy of the surface. However, this also increases the complexity of the fitted surface. Because the established mathematical model was intended for application to the floating plate of a paddy rice transplanter, both fitting accuracy and surface complexity had to be considered during the fitting process. Considering these two factors, the fitting results obtained using different orders of *x* and *y* are presented in Table 1.

**Table 1:** Fitting results of front‐end for different indices of independent variables.

*x* ^*n*^ *y* ^*m*^	SSE	RMSE	*R* ^2^
*xy*	156.9	0.5765	0.9209
*x* ^2^ *y*	135.4	0.5367	0.9318
*xy* ^2^	139.8	0.5453	0.9295
*x* ^2^ *y* ^2^	135.3	0.5371	0.9318
*x* ^3^ *y*	28.9	0.2485	0.9854
*x* ^3^ *y* ^2^	20.99	0.2122	0.9894
*x* ^3^ *y* ^3^	19.82	0.2065	0.99
*xy* ^3^	136.4	0.5398	0.9313
*x* ^2^ *y* ^3^	68.96	0.3847	0.9652
*xy* ^4^	128.9	0.5259	0.935
*x* ^2^ *y* ^4^	61.2	0.3636	0.9692
*x* ^3^ *y* ^4^	17.57	0.1952	0.9911
*x* ^4^ *y*	27.91	0.2447	0.9859
*x* ^4^ *y* ^2^	19.65	0.206	0.9901
*x* ^4^ *y* ^3^	16.63	0.1899	0.9916
*x* ^4^ *y* ^4^	16.54	0.1896	0.9917

*Note*: *x* ^*n*^ *y* ^*m*^ means that the highest index of *x* was *n* and the highest index of *y* was *m* in the fitting equation. SSE is the residual sum of squares, RMSE is root mean square error; the same applies below.

As shown in Table 1, as the polynomial orders of *x* and *y* increase, the three‐dimensional coordinates of the point cloud are progressively better fitted by the polynomial surface in MATLAB. The values of the sum of squared errors (SSEs) and the root mean square error (RMSE) decrease continuously. In contrast, the coefficient of determination (*R* ^2^) increases continuously. These results indicate that, as the polynomial orders of *x* and *y* increase, the fitted surface more closely approximates the topographic characteristics of the actual surface. However, once the polynomial order of *x* reaches 3 and that of *y* reaches 2, the values of SSE, RMSE, and *R* ^2^ change only marginally with each further increase in the order of the independent variables. For example, when the polynomial order increases from *x* ^3^ *y* ^2^ to *x* ^3^ *y* ^3^, SSE decreases from 20.99 to 19.82, RMSE decreases from 0.2122 to 0.2065, and *R* ^2^ increases from 0.9894 to 0.9900. Similarly, when the polynomial order increases from *x* ^3^ *y* ^2^ to *x* ^4^ *y* ^2^, SSE decreases from 20.99 to 19.65, RMSE decreases from 0.2122 to 0.2060, and *R* ^2^ increases from 0.9894 to 0.9901. Moreover, further increasing the polynomial orders yields even smaller changes in these parameters, while substantially increasing the complexity of the fitted surface. When the polynomial order of *x* is 3 and that of *y* is 2, the fitting results are SSE = 20.99, RMSE = 0.2122, and *R* ^2^ = 0.9894, indicating that the coefficient of determination is very close to 1. For the fitting of complex biological surfaces, this level of accuracy satisfies the fitting requirements. The fitted equation for the anterior surface of the turtle shell is as follows:

(1) z=fx,y=2401001226143.82.086−x+y+x2−0.49050.12910.001183xy+y2−x3+0.00041790.0002273x2y−xy2.

**Figure 7:**
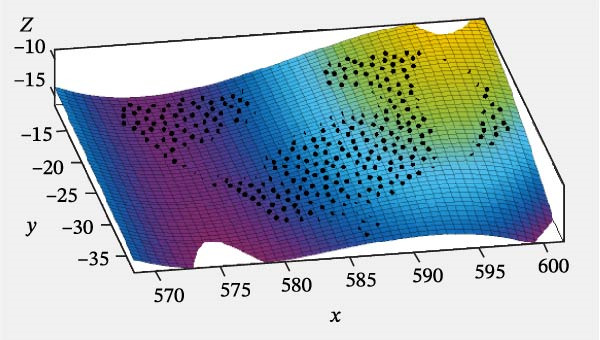
Fitting surface of the front‐end of the turtle shell.

Specifically, the SSE was 20.99, the RMSE was 0.2122, and the coefficient of determination (*R* ^2^) was 0.9894. The MATLAB fitting result of the front‐end surface of the turtle shell is shown in Figure 7.

### 3.2. The Mathematical Model of the Turtle Shell Back‐End Surface

The fitting results obtained using different polynomial orders of *x* and *y* are presented in Table 2, which summarizes the point‐cloud coordinates of the posterior surface in the lower‐left region of the turtle shell.

**Table 2:** Fitting results of the back‐end for different indices of independent variables.

*x* ^*n*^ *y* ^*m*^	SSE	RMSE	*R* ^2^
*xy*	48.43	0.5882	0.7861
*x* ^2^ *y*	23.54	0.4131	0.896
*xy* ^2^	23.97	0.4168	0.8941
*x* ^2^ *y* ^2^	10.77	0.2804	0.9524
*x* ^3^ *y*	20.74	0.3906	0.9084
*x* ^3^ *y* ^2^	3.246	0.1556	0.9857
*x* ^3^ *y* ^3^	2.958	0.1491	0.9869
*xy* ^3^	18.69	0.3707	0.9175
*x* ^2^ *y* ^3^	5.958	0.2109	0.9737
*xy* ^4^	18.01	0.3666	0.9205
*x* ^2^ *y* ^4^	5.421	0.2034	0.9761
*x* ^3^ *y* ^4^	1.941	0.1227	0.9914
*x* ^4^ *y*	20.25	0.3888	0.9106
*x* ^4^ *y* ^2^	1.885	0.1199	0.9917
*x* ^4^ *y* ^3^	1.396	0.104	0.9938
*x* ^4^ *y* ^4^	1.347	0.1026	0.9941

As shown in Table 2, once the polynomial order of *x* reaches 3 and that of *y* reaches 2, the values of SSE, RMSE, and *R* ^2^ change only marginally with any further increase in the order of either independent variable. When the polynomial order increases from *x* ^3^ *y* ^2^ to *x* ^3^ *y* ^3^, SSE decreases from 3.246 to 2.958, RMSE decreases from 0.1556 to 0.1491, and *R* ^2^ increases from 0.9857 to 0.9869. Similarly, when the polynomial order increases from *x* ^3^ *y* ^2^ to *x* ^4^ *y* ^2^, SSE decreases from 3.246 to 1.885, RMSE decreases from 0.1556 to 0.1199, and *R* ^2^ increases from 0.9857 to 0.9917. Furthermore, further increasing the polynomial orders results in even smaller changes in these parameters, while substantially increasing the complexity of the fitted surface. When the polynomial order of *x* is 3 and that of *y* is 2, the fitting results are SSE = 3.246, RMSE = 0.1556, and *R* ^2^ = 0.9857, indicating that the coefficient of determination is close to 1. For the fitting of complex biological surfaces, this level of accuracy satisfies the fitting requirements. In summary, the polynomial order of *x* was selected as 3, and that of *y* was selected as 2. The fitted equation for the posterior surface of the turtle shell is as follows:

(2) z=fx,y=216200132472.342.705−x−y+x2+0.36470.91610.001843xy+y2−x3+0.00044190.00001854x2y−xy2.

**Figure 8:**
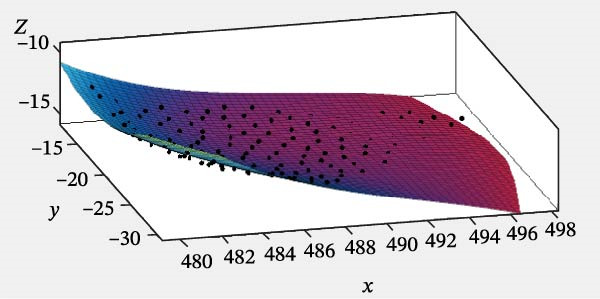
Fitting surface of the back‐end of the turtle shell.

Specifically, the SSE was 3.246, the RMSE was 0.1556, and the coefficient of determination (*R* ^2^) was 0.9857. The MATLAB fitting result for the posterior surface of the turtle shell is shown in Figure 8.

### 3.3. The Mathematical Model of Turtle Shell Side Surface

The fitting results of different indices of x and y are shown in Table 3, which is the point cloud coordinates of the side surface of the turtle shell.

**Table 3:** Fitting results of broadside for different indices independent variables.

*x* ^*n*^ *y* ^*m*^	SSE	RMSE	*R* ^2^
*xy*	1.625e+04	3.458	0.5899
*x* ^2^ *y*	1796	1.15	0.9547
*xy* ^2^	1.532e+04	3.36	0.6134
*x* ^2^ *y* ^2^	1794	1.15	0.9547
*x* ^3^ *y*	1300	0.9794	0.9672
*x* ^3^ *y* ^2^	1223	0.9507	0.9691
*x* ^3^ *y* ^3^	956.9	0.8413	0.9759
*xy* ^3^	1.832e+04	3.194	0.6512
*x* ^2^ *y* ^3^	1037	0.8754	0.9738
*xy* ^4^	1.365e+04	3.176	0.6555
*x* ^2^ *y* ^4^	769.6	0.755	0.9806
*x* ^3^ *y* ^4^	763.9	0.7528	0.9807
*x* ^4^ *y*	1250	0.9611	0.9685
*x* ^4^ *y* ^2^	911.5	0.8217	0.977
*x* ^4^ *y* ^3^	668.7	0.7043	0.9831
*x* ^4^ *y* ^4^	664	0.7021	0.9832

As shown in Table 3, once the polynomial order of *x* reaches 2 and that of *y* reaches 4, the values of SSE, RMSE, and *R* ^2^ change only marginally with any further increase in the order of the independent variable *x*. When the polynomial order increases from *x* ^2^ *y* ^4^ to *x* ^3^ *y* ^4^, SSE decreases from 769.6 to 763.6, RMSE decreases from 0.7550 to 0.7528, and *R* ^2^ increases from 0.9806 to 0.9807. Further increasing the polynomial order of the independent variable *x* results in even smaller changes in these parameters but substantially increases the complexity of the fitted surface. When the polynomial order of *x* is 2 and that of *y* is 4, the fitting results are SSE = 769.6, RMSE = 0.7550, and *R* ^2^ = 0.9806, indicating that the coefficient of determination is close to 1. For the fitting of complex biological surfaces, this level of accuracy satisfies the fitting requirements. In summary, the polynomial order of *x* was selected as 2, and that of *y* was selected as 4. The fitted equation for the lateral surface of the turtle shell is as follows:

(4) z=fx,y=45820169.617850.161+x−y−x2+6.54216.680.006249xy−y2−x3+0.058230.01166x2y−xy2−5.6681.222×10−5x4−×10−5x3y−6.881×10−5x2y2.

**Figure 9:**
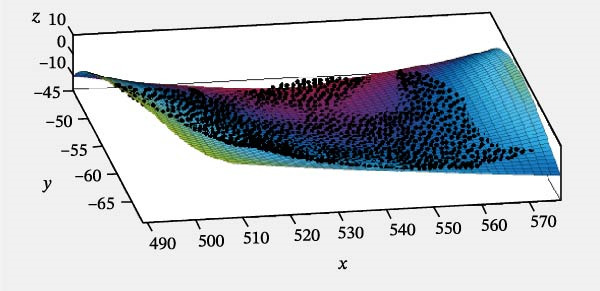
Fitting surface of the side of the turtle shell.

Specifically, the SSE was 769.6, the RMSE was 0.7550, and the coefficient of determination (*R* ^2^) was 0.9806. The MATLAB fitting result for the lateral surface of the turtle shell is shown in Figure 9.

### 3.4. Mathematical Model Verification

To verify the validity of the mathematical model of the turtle shell surface, the point‐cloud data from the right side of the turtle shell in this experiment were used as validation samples. The right side of the turtle shell was likewise divided into the anterior, central, posterior, and lateral regions. Geomagic Studio was used to convert the surface models of these four regions into point‐cloud data, which were then saved as .igs files. The point‐cloud data of the four regions were then imported into the Digitized Shape Editor module of CATIA. The surface point clouds of the four regions were filtered, and the filtered three‐dimensional coordinate values were exported in .txt format. The *x* and *y* values of the point‐cloud data obtained from the four curved surfaces were substituted into the established mathematical model. The fitted values of the mathematical model were then compared with the original coordinate values to determine the residual error range, relative error range, and mean relative error, as presented in Table 4.

**Table 4:** Comparison analysis between fitting values and actual values of four surfaces.

Model	Residual error range	Relative error range (%)	Mean relative error (%)
Front‐end	0.17–5.36	1.48–8.96	4.58
Back‐end	0.12–5.45	0.89–9.36	4.67
Middle	0.26–4.76	1.78–6.32	3.26
Side	0.32–4.38	1.56–5.86	2.98

As shown in Table 4, the calculated values obtained from the four mathematical models for the anterior, central, posterior, and lateral regions of the turtle shell are in good agreement with the measured values, and all relative errors are below 10%. The maximum relative errors for the anterior, central, posterior, and lateral regions were 8.96%, 6.32%, 9.36%, and 5.86%, respectively. The corresponding mean relative errors were 4.58%, 3.26%, 4.67%, and 2.98%, respectively. These mean relative errors satisfy the allowable engineering design error range of ±5%. Therefore, the validity of the four mathematical models for the anterior, central, posterior, and lateral regions of the turtle plastron was verified.

### 3.5. Discussion and Bionic Mapping

The application of such bionic surface modeling in agricultural machinery design carries significant implications [31]. Foremost, it offers a novel solution to reduce mud resistance and soil adhesion on components that operate in wet, sticky environments. Bio‐inspired surface features can dramatically improve performance metrics; adding sinusoidal textures to a soil‐engaging shovel has been shown to keep clay from clinging and to cut fuel consumption by ~17% in high‐moisture conditions [31, 32]. In our context, a turtle‐shell‐inspired floating plate is expected to slide more easily through paddy mud, lowering the drag force and energy required for the transplanter to move forward. Likewise, convex shell and scale motifs applied to rollers and furrow openers have yielded 50% reductions in soil adhesion and significantly improved traction in field tests [33]. These improvements translate to smoother operation, less power slippage, and gentler interaction with the soil and seedlings. In the case of the rice transplanter, reducing the sliding resistance of the floating board via our bionic design could increase transplanting stability and efficiency, minimizing disturbances that can damage young rice plants [34]. The successful mapping of a complex biological surface onto a machine part also highlights the growing synergy between advanced digital design and bionics [35].

This paves the way for more sustainable and efficient agricultural machines that benefit from nature’s time‐tested designs. In summary, by combining polynomial surface modeling with bionic inspiration, we not only achieved a validated mathematical representation of the turtle shell but also provided a clear route to implement this surface on a rice transplanter’s floating plate. The discussion above reinforces that this bionic mapping is both conceptually sound and practically meaningful, yielding a design that is scientifically grounded, engineering‐feasible, and potentially transformative for agricultural machinery performance.

## 4. Conclusion

1. The three‐dimensional point‐cloud data of the Brazilian turtle shell surface were acquired using a 7‐axis Absolute Arm measuring machine. Geomagic Studio was used to process the turtle shell point‐cloud data, including point processing, polygon processing, and precise surface reconstruction. The processing results were evaluated through three‐dimensional deviation analysis. The maximum deviation ranged from −2.4298 to +0.9456 mm, the average deviation ranged from −0.0581 to +0.0730 mm, and the standard deviation was 0.1038 mm.
2. According to the structural characteristics of the turtle shell, it was divided into four curved surfaces, corresponding to the anterior, central, posterior, and lateral regions of the plastron. These four curved surfaces were then fitted individually to obtain the corresponding surface‐fitting equations. The point‐cloud data from the right side of the turtle shell were used as validation samples to verify the four models. The results show that the mean relative errors for the anterior, posterior, and lateral regions were 4.58%, 4.67%, and 2.98%, respectively. These mean relative errors satisfy the allowable engineering design error range of ±5%.

## Author Contributions

Conceptualization, resources: Fu Zhang, Yafei Wang, and Sanling Fu. Methodology, validation, visualization: Zijun Chen and Fu Zhang. Software: Zijun Chen. Formal analysis: Zijun Chen. Investigation: Zijun Chen and Sanling Fu. Data curation: Zijun Chen and Tianhua Chen. Writing – original draft preparation: Sanling Fu, Yafei Wang, and Zijun Chen. Writing – review and editing, supervision: Fu Zhang and Sanling Fu. Project administration, funding acquisition: Fu Zhang.

## Funding

This work was supported by the National Natural Science Foundation of China (Grant 52075149), Frontier Exploration Projects of Longmen Laboratory (Grant LMQYTSKT032), Graduate Education Reform Project of Henan Province (Grant 2023SJGLX180Y), the Scientific and Technological Project of Henan Province (Grant 242102110337), and Colleges and Universities of Henan Province Youth Backbone Teacher Training Program (Grant 2017GGJS062).

## Disclosure

All authors have read and agreed to the published version of the manuscript.

## Conflicts of Interest

The authors declare no conflicts of interest.

## Data Availability

The data used to support the findings of this study are included within the manuscript. Additional data related to this study are available from the corresponding author upon reasonable request.
